# *Myxococcus xanthus* R31 Suppresses Tomato Bacterial Wilt by Inhibiting the Pathogen *Ralstonia solanacearum* With Secreted Proteins

**DOI:** 10.3389/fmicb.2021.801091

**Published:** 2022-02-07

**Authors:** Honghong Dong, Xin Xu, Ruixiang Gao, Yueqiu Li, Anzhang Li, Qing Yao, Honghui Zhu

**Affiliations:** ^1^Key Laboratory of Agricultural Microbiomics and Precision Application – Ministry of Agriculture and Rural Affairs, Guangdong Provincial Key Laboratory of Microbial Culture Collection and Application, State Key Laboratory of Applied Microbiology Southern China, Guangdong Microbial Culture Collection Center (GDMCC), Institute of Microbiology, Guangdong Academy of Sciences, Guangzhou, China; ^2^Guangdong Province Key Laboratory of Microbial Signals and Disease Control, College of Plant Protection, South China Agricultural University, Guangzhou, China; ^3^Guangdong Province Key Laboratory of Microbial Signals and Disease Control, College of Horticulture, South China Agricultural University, Guangzhou, China

**Keywords:** *Myxococcus xanthus* R31, *Ralstonia solanacearum*, predation, biocontrol, extracellular proteins, tomato bacteria wilt

## Abstract

The pathogenic bacterium *Ralstonia solanacearum* caused tomato bacterial wilt (TBW), a destructive soil-borne disease worldwide. There is an urgent need to develop effective control methods. Myxobacteria are microbial predators and are widely distributed in the soil. Compared with other biocontrol bacteria that produce antibacterial substances, the myxobacteria have great potential for biocontrol. This study reports a strain of *Myxococcus xanthus* R31 that exhibits high antagonistic activity to *R. solanacearum*. Plate test indicated that the strain R31 efficiently predated *R. solanacearum.* Pot experiments showed that the biocontrol efficacy of strain R31 against TBW was 81.9%. Further study found that the secreted protein precipitated by ammonium sulfate had significant lytic activity against *R. solanacearum* cells, whereas the ethyl acetate extract of strain R31 had no inhibitory activity against *R. solanacearum*. Substrate spectroscopy assay and liquid chromatography-tandem mass spectrometry (LC-MS/MS) analysis of secreted proteins showed that some peptidases, lipases, and glycoside hydrolases might play important roles and could be potential biocontrol factors involved in predation. The present study reveals for the first time that the use of strain *M. xanthus* R31 as a potential biocontrol agent could efficiently control TBW by predation and secreting extracellular lyase proteins.

## Introduction

Tomato bacterial wilt (TBW) caused by *Ralstonia solanacearum* is a devastating soil-borne disease (Salanoubat et al., 2002). A survey indicated that *R. solanacearum* ranks second among the top 10 most concerned pathogenic bacteria based on the scientific and economic importance (Mansfield et al., 2012). The pathogen *R. solanacearum* is a complex species with obvious physiological differentiation and genetic diversity and a very wide host range. *R. solanacearum* can infect more than 200 species in 54 families of plants (Prior et al., 2016), including tomato, tobacco, potato, banana, pepper, etc., thereby posing a serious threat to food security (Zhang et al., 2020). TBW is a vascular disease that often breaks out under the conditions of high temperatures and high humidity (Choi et al., 2020). Pathogen infection can lead to substantial crop production losses, especially in tropical, subtropical, and other regions with warm temperature (Wang et al., 2018). Now, how to achieve green and efficient control of TBW is a major concern that needs to be solved urgently in agricultural production worldwide.

The traditional methods of controlling soil-borne diseases mainly include physical control and chemical control (Posas et al., 2007). These methods can effectively reduce the number of rhizosphere pathogens and the occurrence of diseases in a short period of time. However, the non-specific bactericidal chemicals not only target the pathogenic bacteria, but also destroy the plant rhizosphere microbial community structure and microecological balance. Consequently, these traditional methods are not conducive for the sustainable development of plant rhizosphere and disease control (Liar et al., 2015). The rhizosphere microbiome is known as the “second genome” of crops and plays a pivotal role in maintaining crop health. Beneficial rhizosphere bacteria-based biocontrol can effectively protect the plants from infection of soil-born pathogenic bacteria, and at the same time can effectively maintain the homeostasis of the rhizosphere microbial community without causing pollution to the environment. Therefore, the use of beneficial rhizosphere microorganisms to control plant diseases has increasingly become a research hotspot and an important direction of applied research (Wei et al., 2018; Elsayed et al., 2020; Im et al., 2020; Ling et al., 2020).

Myxobacteria, as important bacterial predators, are indigenous and dominant bacteria that are widely distributed in the soil (Muñoz-Dorado et al., 2016). It is well documented that myxobacterial isolates can produce rich and diverse secondary metabolites and hydrolytic enzymes, and have broad application prospects in plant disease biocontrol, drug development, waste resources utilization, etc. (Thiery and Kaimer, 2020). In addition, myxobacteria are at the top of the soil microbial food web, and their predation of soil-borne pathogens directly affects the soil microecological environment and plays a pivotal role in maintaining the soil microecological balance and plant health (Marshall and Whitworth, 2019). Moreover, myxobacteria have high abundance in the soil, strong resistance to stresses, high potential for producing active substances, and a wide range of predation. These characteristics endow the myxobacteria with unique biocontrol advantages.

In recent years, numerous researches have suggested myxobacteria to be a type of promising biocontrol agent with higher potentials to inhibit the agricultural pathogen. Some greenhouse experiments and field trials determine that the application of myxobacteria remarkably alleviates damping-off disease of tree seedlings (Hocking and Cook, 1972; Dahm et al., 2015), cucumber Fusarium wilt (Löbmann et al., 2016; Ye et al., 2020), hot peppers anthracnose (Kim and Yun, 2011; Raza et al., 2017), and rice blast (Li et al., 2017). Intriguingly, most studies report the biocontrol potentials of myxobacteria against a variety of plant fungal diseases rather than bacterial diseases (Homma, 1984; Iizuka et al., 1998; Taylor and Draughon, 2001; Bull et al., 2002). For example, myxobacteria *Corallococcus* sp. EGB secretes a new type of β-1,6-glucanase GluM, which specifically targets β-1,6-glucan in the cell wall of phytopathogenic fungi by destroying the integrity of the cell wall, and thus inhibits fungal infection (Li et al., 2019b). Strain EGB also secretes a chitin hydrolase, CcCti1, which exerts an antibacterial effect by inhibiting the germination of *Magnaporthe oryzae* and the formation of appressorium (Li et al., 2019a). In contrast, only a few experiments report the application of myxobacteria in the biocontrol of bacterial diseases, where *Myxococcus* sp. strain BS effectively reduced the incidence of calla lily soft rot caused by *Pectobacterium carotovorum* (Li et al., 2018). Recently, we found that myxobacteria could efficiently predate *R. solanacearum*, the primary bacterial pathogen of TBW, in laboratory assays. In this scenario, we propose that myxobacteria may be developed as a robust biocontrol agent to suppress TBW, and the predation mechanisms of myxobacteria on the phytopathogenic bacterium *R. solanacearum* deserve further elucidation.

The present study aimed to isolate and screen myxobacterial isolates that can effectively control TBW and to explore the underlying biocontrol mechanisms. Here, by using *E. coil* and *R. solanacearum* as the prey bacteria, we isolated fifty myxobacteria from the healthy tomato rhizosphere soil of the TBW field. With the combination of plate predation assay and pot experiments, we found that *M. xanthus* R31 not only effectively predated *R. solanacearum* on plates, but also exhibited an excellent biocontrol potential against TBW in pot experiments. Further studies indicated that the extracellular enzyme proteins, especially peptidase, lipases, and glycoside hydrolases secreted by strain R31 played a significant role in the predation process. The present study provides a new insight into the biocontrol against TBW and the recognition of myxobacteria predation against phytopathogen bacteria.

## Materials and Methods

### Strains and Culture Conditions

The phytopathogen bacterial strains *R. solanacearum* RsH, GIM 1.70, RS04, GMI 1000, GIM 1.335, and CFP-tagged RsH were maintained in our laboratory under −80°C with 25% glycerol as the cryoprotectant. Strains were grown on triphenyl tetrazolium chloride (TTC) solid medium (Popoola et al., 2015) at 30°C. When necessary, gentamycin (Gm) at a final concentration of 30 μg mL^–1^ was added to the culture medium. For pathogen infection assay, a single colony of RsH was inoculated into TTC liquid broth and cultured in a horizontal shaker at 30°C with 200 rpm for 2 days. Myxobacteria strains and the new isolates were cultured on Casitone-Tris (CTT) (Nair et al., 2019) or VY/4 medium (Li et al., 2017) at 28°C. *Escherichia coli* 1.173 was grown at 37°C in LB broth.

### Isolation of Myxobacteria in the Soil of the Diseased Area

Soil samples were collected from the experiment field (N23°9′44″, E113°22′22″) of South China Agricultural University, Guangzhou, China. Approximately 200 g of soil was collected from the upper 5–15 cm layer. The samples were air-dried as quickly as possible after collection and stored at room temperature after passing through a 2-mm pore-sized mesh. The isolation procedure of myxobacteria with the induction of fruiting body formation was performed as described before with minor revision (Iizuka et al., 1998). Briefly, a sterile needle was used to pick the fruiting bodies that were induced by *R. solanacearum* RsH or *E. coil* 1.173, and then the fruiting bodies were transferred to VY/4 purifying medium and cultured at 28°C for 3–7 days, and this step was repeated for several times until no other bacterial taxa grew.

### Identification of the Myxobacterium Isolates

The purified myxobacterial isolates were inoculated on VY/4 medium for morphological observation. For the phylogenetic analysis, the 16S rRNA gene was amplified using the primers 27F (5′-AGAGTTTGATCCTGGCTCAG-3′) and 1492R (5′-GGTTACCTTGTTACGACTT-3′) (Weisburg et al., 1991). The PCR amplification was conducted in a T100™ PCR system (Bio-Rad, Hercules, CA, United States) using *EasyTaq* DNA polymerase (Transgene, China) with following conditions: 94°C for 5 min, followed by 35 cycles at 94°C for 15 s, 58°C for 30 s, and 72°C for 2 min, and a final extension at 72°C for 5 min. PCR amplification products were detected using 1% agarose gel electrophoresis analysis and sequenced by Shanghai Sangon Biotechnology Co., Ltd. (Shanghai, China). The similarity search of the 16S rRNA gene was performed using EzBioCloud database.^1^

### Predation Activity Assay

Predation activity was assayed using the colony-induced predation method as described (Berleman et al., 2006). Firstly, the predation ability of the isolated myxobacteria strains against *R. solanacearum* on the TPM plate was estimated to screen the strains with strong predation ability for further research. A total of 100 μL RsH cell suspension was pipetted onto the TPM plates and allowed to dry, and then 4 μL myxobacterial suspension was spotted at a 2 mm distance from the prey colony. Plates were cultured at 28°C for 7 days. The lawn growths were examined under a stereoscope and photographed. Here, strain of *M. xanthus* R31 displayed a high predation activity against *R. solanacearum*. Therefore, predation activity of strain R31 was further evaluated on WA (No nutrition) and CFL (Oligotrophic) plates with the same methods as above. Predator area rate which was evaluated based on its swarming area against the pathogenic lawn area, and the numbers of fruiting bodies were used to quantify the predation efficacy of strain R31. Additionally, strain R31 and RsH were co-cultured on CFL plate as described above, and the bacterial lawn comprising both of strain R31 and RsH was taken for scanning electron microscope (SEM, Hitachi S-3000N) examination.

### Pot Experiments With Tomato Bacterial Wilt

The field soil grown with tomato all year round was used as the substrate in the pot experiments. The soil was air-dried soon after collection, screened through a 2-mm pore-sized mesh, and stored at room temperature. A susceptible tomato cultivar Zhongshu No. 4 was used as the test plant. Tomato plants were grown in a greenhouse under 80% relative humidity at 28°C and in a 16 h/8 h light/dark cycle.

Three treatments were designed: (1) only pot soil used as control; (2) pot soil inoculated with RsH at 1 × 10^7^ colony-forming units (CFU) g^–1^ soil; and (3) pot soil simultaneous inoculated with strains R31 (5 × 10^5^ cells/mL) and RsH (1 × 10^7^ CFU g^–1^ soil). One tomato plant was grown in each pot, and each treatment included six pots representing six biological replicates. The experiments were repeated three times.

The typical wilt symptoms of TBW were evaluated in terms of five disease severity scores from 0 to 4, where 0 represents no symptoms, and 1, 2, 3, 4 represent <25, 26–50, >50, and 100% of leaves being wilted, respectively. The disease index (DI) and biocontrol efficacy were subsequently calculated as following: DI = [∑(number of diseased plants × disease degree)/(totalnumber of plants × highest disease degree)] × 100. Biocontrol efficacy = (control disease index − treatment disease index)/control disease index × 100% (Wang et al., 2019).

### Real-Time Quantitative PCR Analysis

The abundances of *R. solanacearum* RsH in the rhizosphere soil, tomato root, and stem tissues were determined by RT-qPCR quantification of the *filC* gene. RT-qPCR was performed with an Applied Biosystems Quant Studio 6 and 7 Flex Real-Time PCR system (Applied Biosystems, Foster City, CA, United States) using a TransStart Tip Green qPCR Super Mix (Transgene, China). The primers filC-F (5′-GAACGCCAACGGTGCGAACT-3′) and filC-R (5′-GGCGGCCTTCAGGGAGGTC-3′) were used for the detection of the abundance of RsH (Schönfeld et al., 2003).

### Confocal Laser Scanning Microscopy Observation

Colonization of *R. solanacearum* RsH in roots and stems of tomato inoculated with CFP-tagged RsH or strain R31 + CFP-tagged RsH were examined using confocal observation. Microscope observation of the sliced plant tissues was performed under a confocal Laster-scanning microscope (LSM 700, Zeiss) equipped with filter blocks with spectral properties matching the fluorescence of CFP (excitation wavelength at 405 nm and emission wavelength at 485 nm) and the autofluorescence of tomato tissues (excitation wavelength at 543 nm and emission wavelength at 590 nm).

### Evaluation of Secondary Metabolites and Extracellular Proteins of Strain R31 in the Predation Against RsH

Strain R31 was cultured in CTT liquid for 7 days at 30°C and shaking at 160 rpm. The culture suspension was collected by centrifugation at 8,000*g*, and secondary metabolites were extracted from the supernatant with an equal volume of ethyl acetate, whereas the intracellular metabolites were extracted after the cells were ultrasonically broken up. The extracts were dissolved in an appropriate volume of methanol to a final concentration of 50 mg mL^–1^. For bacteriostatic assay, 30 μL methanol extract was added to a circular filter paper (6 mm diameter), and after methanol was completely removed by evaporation, the filter paper was overlaid on TTC medium spread with strain RsH. Methanol and Gm were used as the negative control and positive control, respectively. The inhibitory activity of ethyl acetate extract against RsH was determined according to the inhibitory zone size.

To investigate the lysis activity of extracellular proteins of strain R31 against RsH, strain R31 was cultured in CTT liquid medium at 30°C for 3 days with shaking at 180 rpm, and the spent culture was collected by centrifugation at 12,000*g*. Protein in the spent culture was precipitated with ammonium sulfate at various saturations (Li et al., 2019b), dissolved in PBS buffer (0.01 M, pH 7.2), and then dialyzed in a 3.5 kDa molecular weight dialysis bag to remove the residual ammonium sulfate. The dialyzate was then concentrated with an Amicon ultrafiltration tube of 3 kDa (Millipore, United States) and added with the newly cultured RsH cells. After incubation at 37°C for 2 h, the cell integrity of strain RsH cells was examined under a transmission electron microscope (TEM, Hitachi H-7650). The heat-inactivated protein solution was used as a blank control. For cell viability of RsH detection, extracellular proteins of strain R31 and newly cultured RsH cells were incubated at 37°C for 0, 3, 5, 8, and 12 h, respectively. Then, the plate gradient dilution method was used to calculate the numbers of live RsH cells.

### Substrate Spectrum Analysis of Extracellular Enzyme Crude

Laminarin and carboxymethyl cellulose were used as the substrates to assay the polysaccharide lyase activity in the extracellular protein. The enzyme activity was determined with 3,5-dinitrosalicylic acid (DNS) method using DNS assay kits (Beijing Solarbio Science Technology Co., Ltd., China) according to the manufacturer’s protocol. Each experiment was repeated three times. The 4-nitrophenyl octanoate and *p*-nitrophenyl palmitate were used as substrates for lipase activity assay by spectrophotometry, as described by Zheng et al. (2011), and a series diluted *p*-nitrophenol was used to produce the standard curve. Inactivated crude enzyme solution was used as a negative control, and each test was repeated three times.

### Liquid Chromatography-Tandem Mass Spectrometry Analysis of Extracellular Proteins With Lysis Activity

Proteins in 40% saturated ammonium sulfate precipitation were reduced with 0.05 M Tris (2-carboxyethyl)phosphine (TCEP) for 1 h at 60°C. The protein was alkylated with 55 mM methyl methanethiosulfonate (MMTS) for 45 min at room temperature in darkness, then added into 10 K Millipore, centrifuged at 12,000*g* at 4°C for 20 min, and the filtrate was discarded. One hundred microliters of UA buffer (8 M urea, 0.1 M Tris–HCl, pH 8.5) was added into Millipore and centrifuged at 12,000*g* at 4°C for 20 min two times, and the filtrate was discarded. Then 100 μL 0.25 M triethylammonium bicarbonate (TEAB) was added into the Millipore and centrifuged at 12,000*g* at 4°C for 20 min three times, and the filtrate was discarded. Trypsin was added at 1:50 trypsin-to-protein mass ratio for the first digestion overnight and 1:100 trypsin-to-protein mass ratio for a second 4 h-digestion. The mixture was centrifuged at 12,000*g* at 4°C for 20 min. The filtrate was collected, added with 50 μL of 0.5 M TEAB, then centrifuged at 12,000*g* for 10 min, the filtrate was collected and vacuum dried at low-temperature until LC-MS/MS analysis.

In this study, LC-MS/MS analysis was entrusted to Guangzhou FitGene Biotechnology Co., Ltd. (Guangzhou, China) to be completed, and the specific experimental procedures are shown in Supplementary File 1. Finally, protein identification was performed with Mascot search engine (v2.3.0) by searching strain R31 protein databases.^2^

## Results

### Myxobacteria Isolates From the Healthy Tomato Rhizosphere Soil of the Tomato Bacterial Wilt Field Display Predatory Activity on RsH

**FIGURE 1:**
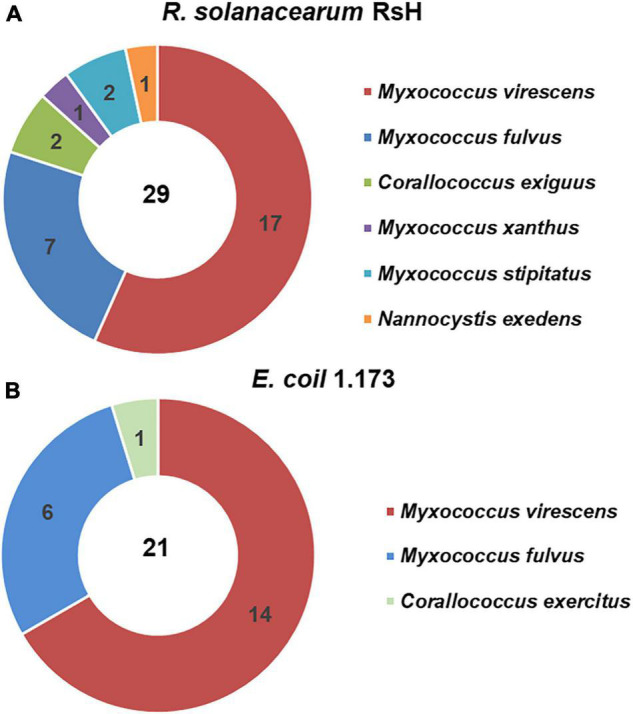
Isolated myxobacteria species from the healthy tomato rhizosphere soil of the tomato bacteria wilt (TBW) field. Using *Ralstonia solanacearum* RsH **(A)** and *Escherichia coil* 1.173 **(B)** as the prey bacteria, the myxobacteria strains were isolated from the TBW soil sample. The 50 isolates were identified based on the 16S rRNA gene identity analysis. The pie charts showed the species and numbers of the myxobacteria isolates using the two prey bacteria.

To obtain the potential biocontrol agent of myxobacteria to suppress TBW, we used *E. coli* 1.173 and *R. solanacearum* RsH as the prey bacteria and isolated 50 myxobacteria strains (Supplementary Table 1). Based on the 16S rRNA gene sequence homology analysis, the 50 myxobacterial strains were identified as three genera and seven species were affiliated to Myxococcaceae and Nannocystaceae families. Six myxobacterial species were isolated using *R. solanacearum* RsH as prey, in contrast to only three using *E. coli* 1.173 as prey, although similar numbers of the myxobacteria isolates with the same representative species (*M. virescens and M. fulvus*) were obtained using two different prey species (Figure 1). This probably implies that the phytopathogen *R. solanacearum* could be a preferential prey of myxobacteria.

**FIGURE 2:**
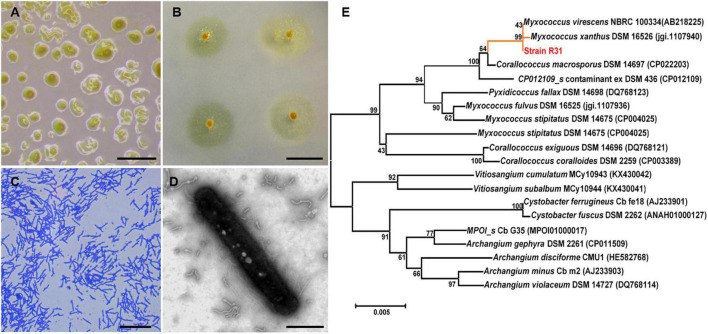
Morphology and phylogenetic identification of the isolated *Myxococcus xanthus* R31. **(A)** Fruiting body formation of strain R31, scar bar = 0.5 mm; **(B)** Swarming growth of strain R31 on VY/4 medium, scar bar = 1 cm; **(C)** Micrograph of strain R31, scar bar = 15 μm; **(D)** transmission electron microscopy (TEM) micrograph of strain R31, scar bar = 5 μm; **(E)** Neighbor-joining tree of strain R31 based on 16S rRNA gene sequence.

Next, we evaluated the predatory activity of the myxobacteria isolates against *R. solanacearum* using plate experiment. Excitingly, *M. xanthus* R31 displayed a high predation activity against all five tested *R. solanacearum* strains (Figure 2 and Supplementary Figure 1). Within 7 days of co-culture, strain R31 swarmed into a large part of lawns of the prey *R. solanacearum* (Supplementary Figure 2). This indicates that strain R31 could be potential to suppress *R. solanacearum*.

### Strain R31 Predates and Breaks Up the Prey RsH Cells

**FIGURE 3:**
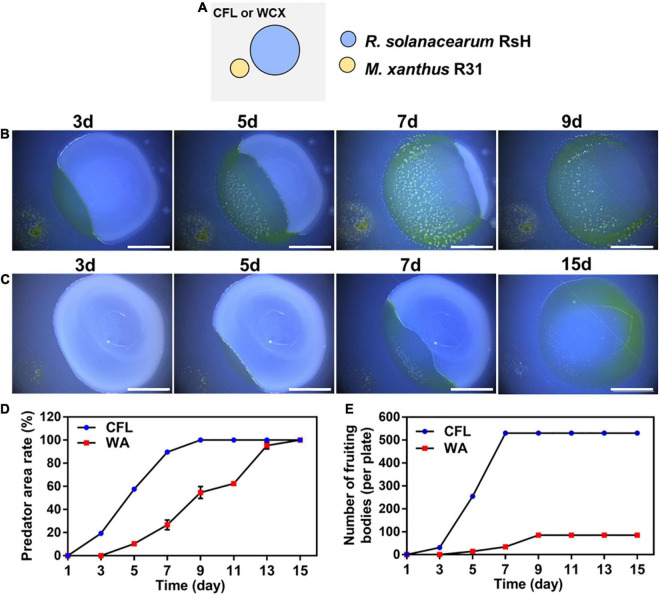
Predation activity of strain R31 against RsH on CFL and WA media. Schematic of the predation assay **(A)**, 100 μL RsH cell suspension was pipetted onto the CFL **(B)** and WA plates **(C)** and allowed to dry, and then 4 μL of strain R31 suspension was spotted at 2 mm distance from the prey colony, scar bar = 5 mm; Predator area ratio on two different media **(D)** and the numbers of strain R31 fruiting bodies formed on two media **(E)** was calculated. Triplicate experiments were performed, and the averages and standard deviations are shown.

To further estimate the predatory ability of strain R31 in various environments, two media, CFL (Oligotrophic), and WA (No nutrition) were used. In co-culture of strain R31 and the RsH, swarming growth of strain R31 occurred in the two media (Figure 3). However, the active swarming growth of strain R31 toward RsH was faster on CFL medium than that on WA medium. Specifically, strain R31 almost completely extended into the prey lawn within 7 days co-culture, whereas it took 15 days for strain R31 on WA medium (Figures 3B,C). Calculation indicates that strain R31 exhibited a higher predatory activity on CFL medium than on WA medium (Figures 3D,E). In addition, more fruiting bodies were observed for strain R31 on CFL medium compared with WA medium (Figure 3E).

**FIGURE 4:**
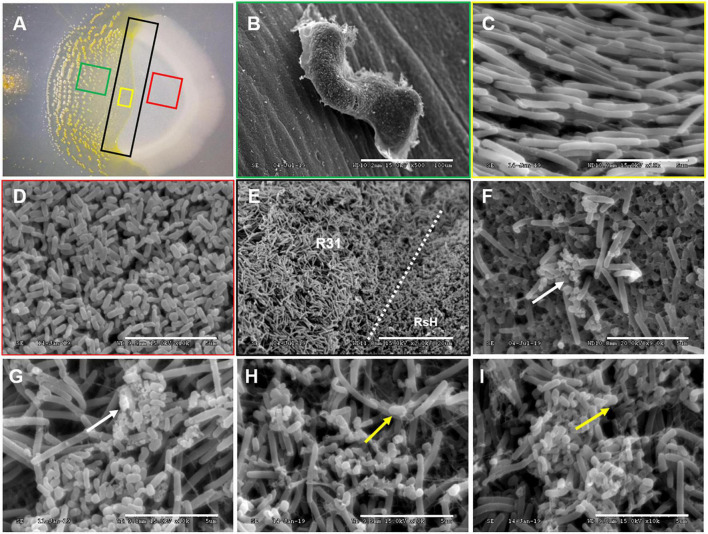
SEM demonstrating the predation of RsH by strain R31. Taking a sample of the strain R31 predation on RsH in CFL plate **(A)** for scanning electron microscope (SEM) observation, **(B)** strain R31 that have completed predation in panel **(A)** green box formed a clear fruit body structure, scar bar = 100 μm, **(C)** strain R31 preparing to predation in panel **(A)** yellow box showed tropism, scar bar = 5 μm, **(D)** normal RsH cell was densely distributed with complete morphological structure, scar bar = 5 μm, **(E)** the boundary line where strain R31 predation on RsH, strain R31, and RsH exist at the same time, and strain R31 swarmed in the direction of the RsH cells during predation, scar bar = 20 μm, **(F–I)** strain R31 came into contact with RsH cells, the morphological structure of RsH cell is destroyed, and the cells were broken into small pieces. The white arrows indicated the *R solanacearum* cells whose cell structure has been destroyed. The yellow arrows indicated that strain R31 secretes a large amount of filamentous extracellular substance entangled RsH cells, scar bar = 5 μm.

Scanning electron microscopy was further used *in situ* to observe the predation of RsH cells by strain R31 (Figure 4A). The strain R31 that have finished predation formed a clear fruit body structure (Figure 4B), but swarmed in the direction of the RsH cells during the predation (Figure 4C). RsH cells were densely clustered with complete morphological structure before being predated by strain R31 (Figure 4D). Once the strain R31 came into contact with and predated the RsH cells, the morphological structure of the RsH cells was destroyed and the cells were broken into small pieces (Figures 4F–I). Interestingly, the destroyed RsH cells were surrounded by many filamentous substances (Figure 4F), which might be some extracellular material secreted by strain R31 to lyse the RsH cells.

### Strain R31 Increases Tomatoes Resistance Against Tomato Bacterial Wilt by Decreasing the Abundance of RsH

**FIGURE 5:**
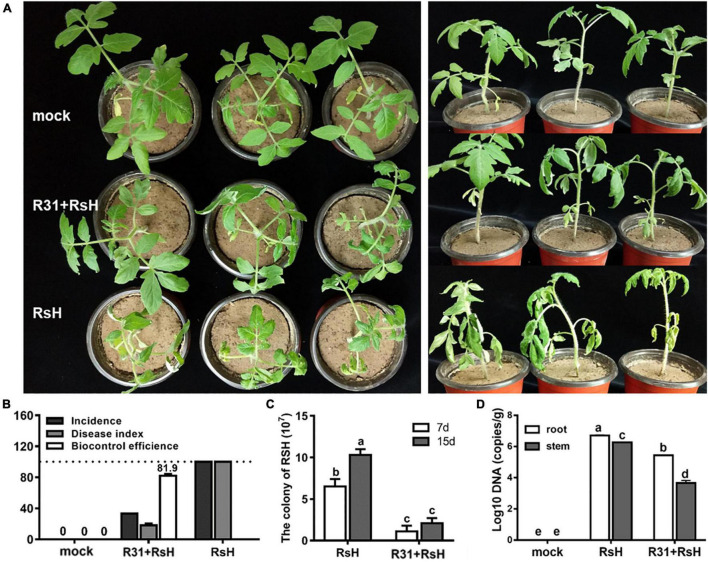
Pot experiments assayed strain R31 suppressing TBW. **(A)** Greenhouse pot experiments of strain R31 for biocontrol of TBW, mock was a sterile water control, R31 + RsH was for simultaneous inoculated of strain R31 and RsH, and RsH was only inoculated with RsH. After continued cultivation for another 7 days, the treatments were photographed. The left and right panels show the top and the side views, respectively. **(B)** Biocontrol effect statistics of strain R31 against TBW, plant disease status was evaluated based on equation that is described in the “Materials and Methods” section. **(C)** The abundance of RsH was tested by counting the colony formation units. Each 10 g of soil was sampled from the tomato rhizospheres that were added only with RsH (RsH) and with strain R31 and RsH (R31 + RsH), respectively. After a 10-fold series dilution, the soil suspension was spread on TTC plate. Colony was counted after 2 days incubation. **(D)** Quantitative results of RsH in tomato root and stem tissues were obtained through RT-qPCR, uninoculated tomato root and stem tissues as a mock. Triplicate experiments were carried out, and the averages and standard deviations are shown. The lowercase letters at each bar top show the statistically significant differences by Duncan’s test (*p* < 0.05).

We conducted greenhouse pot experiments to verify the biocontrol potential of strain R31. The result showed that the tomato plants simultaneously inoculated with strain R31 and RsH almost grew as healthy as the control plants, whereas the plants inoculated with only RsH were seriously ill or even died (Figure 5A). Calculation indicates that the biocontrol efficacy of strain R31 against TBW was 81.9%, and the DI decreased from 100 in the R31 + RsH treatment to 18 in the RsH treatment (Figure 5B).

Furthermore, we analyzed the abundance of RsH in rhizosphere soil, tomato root and stem tissues, respectively, and found that strain R31 significantly decreased the abundance of RsH in soil and plant tissues (Figures 5C,D). Histological observation assay showed a similar result (Supplementary Figure 3). Collectively, these results indicate that strain R31 increased tomato resistance against TBW by decreasing the abundance of RsH.

### Extracts of Strain R31 Fermentation Broth Have No Antibacterial Activity Against RsH

Since secondary metabolites play a pivotal role during the predation event of myxobacteria, we extracted the secondary metabolites in fermentation supernatant and bacterial cells of strain R31 using the ethyl acetate. The LC-MS analysis of strain R31 fermentation supernatant and the extracts in the bacterial cells revealed that the kind of substances extracted from the fermentation supernatant was more than that extracted from the bacterial cells (Supplementary Figure 4). Antibacterial activity test shows that compared with the positive control, the ethyl acetate extract from strain R31 cells and supernatant had no inhibitory activity against RsH (Supplementary Figure 5). Therefore, we speculated that the strain R31 predation on RsH might not be attributed to the secondary metabolites.

### Extracellular Proteins of Strain R31 Efficiently Lyse RsH Cells

**FIGURE 6:**
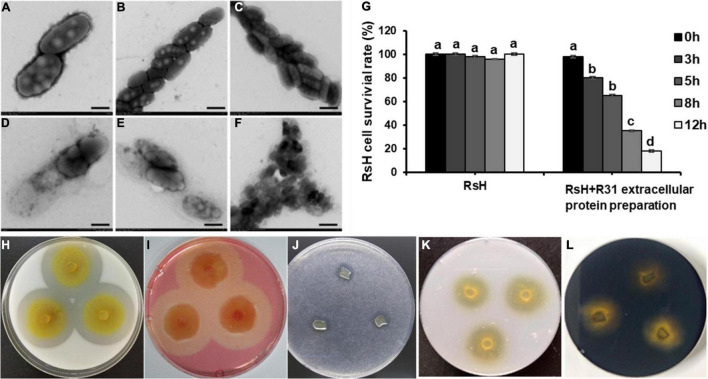
Lysis of RsH by strain R31 extracellular proteins and assay of strain R31 degradation of various polymers. TEM micrograph of RsH cell after treatment with PBS buffer control **(A–C)** and extracellular proteins **(D–F)**. Scar bar = 50 nm for panels **(A,D)**, scar bar = 1 μm for panels **(B,C,E,F)**. **(G)** Statistical analysis of cell viability of RsH treated with strain R31 extracellular proteins at different times. Error bars represent standard errors (±SD) from three repeated experiments. Duncan multiple comparison test (*p* < 0.05) was performed on the data, the same letter means no difference, and different letters mean there is a difference. For panels **(H–L)**, skimmed milk **(H)**, sodium carboxymethyl cellulose **(I)**, tributyrin **(J)**, colloidal chitin **(K)** and starch **(L)** were each added to CTT plate at a final concentration of 0.5%. The exponential culture of strain R31 was spotted and incubated at 30°C. After 4 days of incubation, the plates were photographed. Triplicated experiments were performed for each test, and the representative images are shown.

We extracted the extracellular proteins of the strain R31 to determine their lytic bacteria ability against RsH cells. Excitingly, compared with the control (Figures 6A–C), we found that the protein components were precipitated with 40% saturation ammonium sulfate, showing a significant lysis effect on RsH cells (Figures 6D–F). The structure of RsH cells became loose and irregular, cell contents overflowed, and the integrity was destroyed (Figures 6D–F), whereas the protein components precipitated by saturated ammonium sulfate of other concentrations (40–60, 70–80, and 80–100%) showed no activity (Supplementary Figure 6). These results indicate that the effective proteins might exist in the fraction precipitated by 40% saturation ammonium sulfate. The cell viability of RsH before and after treatment with the extracellular protein was further tested using the method of plate gradient dilution, and the result showed that the treatment with the extracellular proteins of strain R31 significantly reduced the cell viability of RsH along with the time (Figure 6G).

We tested the lytic activity of strain R31 on different substrates. The results showed that strain R31 degraded skimmed milk, sodium carboxymethyl cellulose, and tributyrin (Figures 6H–J), but did not degrade chitin and starch (Figures 6K,L).

Next, we further analyzed the substrate spectrum of the extracellular proteins fraction with lysis effect. Laminarin with β-1,6 glycosidic bonds and carboxymethyl cellulose with β-1,4 glycosidic bonds were hydrolyzed by the extracellular proteins, and the lipase substrates 4-nitrophenyl octanoate and *p*-nitrophenyl palmitate were also effectively hydrolyzed (Table 1). In contrast, the extracellular proteins showed no active reaction to D-glucan with β-1,3-glycosidic bonds and xylan. Therefore, it is possible that the extracellular proteins of strain R31 might have the lipase activity, the cellulase activity, and the glycoside hydrolase activity of hydrolyzing β-1,6-glycosidic bonds.

**TABLE 1:** Substrate specificity of extracellular crude enzymes of strain R31.

Substrate	Bond types	Enzyme activity
Xylan	β-1,4-(Xylopyranosyl)	0
D-Glucan	β-1,3 (Glycose)	0
Chitin	β-1, 4-*N*-Acetylaminoglycoside bond	0
Laminarin	β-1,3-β-1,6-(Glucose)	4.296 ± 0.021 U mL^–1^
Carboxymethyl cellulose	β-1,4 (Glycose)	0.252 ± 0.009 U mL^–1^
*p*-Nitrophenyl palmitate	Ester linkage	0.163 ± 0.008 U mL^–1^

The LC-MS/MS analysis revealed a total of 178 proteins in the extracellular proteins precipitated with 40% saturation ammonium sulfate (Supplementary Table 2). Among them, we identified 9 enzymes with lytic activity, including one M4 family metalloprotease, three peptidase, one endopeptidase, two glycoside hydrolases, one esterase, and one lipase. To sum up, these results suggest that these extracellular enzyme proteins may play a significant role in the predation of strain R31.

## Discussion

In recent years, biocontrol agents based on beneficial soil microorganisms have attracted the attention of scientists to control TBW and have also achieved certain results. In this study, we successfully isolated and screened a strain R31 for potential use as an effective biocontrol agent against TBW and showed that it effectively suppressed this disease in pot experiment. While many species have been used for biological control of TBW, including *Streptomyces* (Ling et al., 2020), *Bacillus methylotrophicus* (Im et al., 2020), *Bacillus amyloliquefaciens* (Wei et al., 2011; Ho et al., 2020), *Bacillus velezensis* (Chen et al., 2020), *Streptomyces microflavus* (Shen et al., 2021), information on the use of myxobacteria as biocontrol agents in controlling TBW is blank. In fact, myxobacteria have great potential in biocontrol of plant diseases. Antifungal myxobacteria, such as *Corallococcus* (Li et al., 2017; Ye et al., 2020), *Myxococcus* (Kim and Yun, 2011), *Sorangiym cellulosum* (Hocking and Cook, 1972), *Nannocystis exedens* (Taylor and Draughon, 2001), and also other predatory myxobacteria (Homma, 1984; Meliah et al., 2020), have shown good biocontrol effects on a variety of plant fungal diseases. Actually, myxobacteria have stronger ability to prey on bacteria, and their potential for biocontrol of plant bacterial diseases is more promising. *Myxococcus* sp. strain BS as an efficient biocontrol agent for soft rot of calla lily (Li et al., 2018) is a good example. To the best of our knowledge, our study is the first report demonstrating the biocontrol of TBW by myxobacteria in pot experiments.

*Ralstonia solanacearum* is a soil-borne pathogen, which is extremely difficult to prevent and control due to the high degree of adaptability and variability (Genin and Denny, 2012). While the predation range of myxobacteria is very wide, and its preference for prey bacteria is generally reflected in the level of large taxonomic units such as phylum and class, the physiological differentiation of the same bacteria is normally ignored (Morgan et al., 2010). In the present study, we found that strain R31 showed efficient predation on five strains of *R. solanacearum*. Therefore, we speculate that myxobacteria have an important biocontrol significance for TBW.

As a model species, *M. xanthus* is often used to study the multicellular behavior of myxobacteria (Muñoz-Dorado et al., 2016). Previous research has shown that in the process of predation, myxobacteria can secrete a large number of hydrolytic enzymes to kill and decompose prey cells and release the hydrolyzate into the extracellular environment, which is consumed by it for growth (Evans et al., 2012). Myxobacteria can produce a variety of enzymes, including proteases, amylases, cellulases, lipases, chitinases, xylanase enzymes, etc., which are the material basis for their predatory ability (Muñoz-Dorado et al., 2016). Myxobacteria can predate a variety of prey microorganisms through their unique wolf-pack behavior. The hypothesis is that when the cell density is high, the myxobacteria secrete a variety of hydrolytic enzymes. Myxobacterial cells gather together in the external environment to increase the concentration of enzymes and jointly play a lysis effect, creating a shared pool of hydrolyzates, which is convenient for individual cells to absorb the lysates to achieve growth and reproduction (Berleman and Kirby, 2009). Recently, Li et al. (2017) suggested that the extracellular supernatant of strain EGB effectively reduced the infection ability of the *M. oryzae* on rice seedlings. The authors isolated a β-1,6-glucanase (GluM) and confirmed that it played a role in lysing fungal cell walls in the process of myxobacteria preying on fungi (Li et al., 2019b). Myxobacteria can use lipolytic enzymes to remove the cell membrane barrier of the prey and empty the cytoplasmic content of the prey (Moraleda-Munñoz and Shimkets, 2007). In line with these results, we also identified some enzyme proteins in strain R31 extracellular proteins, which have a lysis activity against RsH. Therefore, we speculate that the extracellular enzyme proteins, especially some peptidase, lipase, glycoside hydrolases etc., have a significant role in the predation of strain R31 against *R. solanacearum* and thus in the biocontrol of TBW. Certainly, further study to verify these speculations is necessary.

Secondary metabolites produced by myxobacteria are considered as small-molecule weapons that can penetrate prey cells, stop metabolism, or kill them (Pérez et al., 2020). In *M. xanthus* DKI622, the antibiotic TA has been proven to inhibit the growth of *E. coli* MG1655, but has no inhibitory effect on the growth of the gram-positive bacterium *Micrococcus luteus*, indicating that TA shows selective activity against bacterial species (Goldman et al., 2006; Xiao et al., 2011). Moreover, the killing ability of TA depends on the metabolic activity of the prey cells. The production of TA is very important for killing the metabolically vigorous and growing *E. coli*. In this study, however, we found that small molecules extracted with ethyl acetate had no antibacterial activity on RsH. Therefore, we inferred that the secondary metabolites produced by strain R31 may not play a role in the biocontrol of TBW. Similarly, Li et al. (2017) found that the secondary metabolites secreted by the *Corallococcus* sp. EGB had no antibacterial activity against phytopathogenic fungi. Interestingly, we found that *Citreicoccus inhibens* gen. nov. sp. nov. M34 suppressed or predated a variety of phytopathogen mainly by secreting secondary metabolisms (Zhou et al., 2021). Generally speaking, we speculate that this may be related to the differences between different species in the coevolution process of myxobacteria, plants, and microorganisms.

Predation is an important survival strategy for most myxobacteria. The predation of myxobacteria on gram-negative and gram-positive bacteria is mediated by different bactericidal mechanisms (Arend et al., 2021). During the predation of fungi by *Corallococcus* sp. EGB, the strain EGB destroyed the fungi cell wall by secreting GluM and CcCti1 (Li et al., 2019a,b). In the preset study, we found that peptidase, lipases, and glycoside hydrolases played a key role during the predation of strain R31 against *R. solanacearum*. These results might suggest that the kind of enzymes secreted by myxobacteria to lyse prey cells during predation is probably related to the composition of the cell wall of prey cells. Certainly, the types of lytic enzymes secreted by myxobacteria during predation and their mode of action on prey cells need further research. More detailed transcriptome and proteomes sequencing data, and also the analysis of the interaction between myxobacteria, prey, and plants will be expected to help identify specific extracellular lyase species involved in predation and to clarify the predation mechanism of myxobacteria in the future.

Rhizosphere exudates cannot only provide nutrients for indigenous microorganisms, but can also be used by plants to attract or repel related microorganisms (Dayakar et al., 2009). Ye et al. (2020) reported that the myxobacteria strain EGB responds chemotaxis to cucumber root exudates. In this study, strain R31 showed a good biocontrol effect on TBW. Therefore, it is necessary to further study how tomato root exudates affect the interaction between strain R31 and *R. solanacearu*. It helps to better apply myxobacteria to the biocontrol of TBW.

## Conclusion

In this study, we successfully isolated fifty myxobacteria strains from the healthy tomato rhizosphere soil of the TBW field. We showed that myxobacteria strain R31 can be used as a potential biocontrol agent of TBW. The strain R31 exhibited efficient predation against *R. solanacearum* in the plate assay and effectively reduced the abundance of *R. solanacearum* in the pot experiment, which was significant to ensure an effective biocontrol of TBW. In addition, our results also indicate that the extracellular enzyme proteins secreted by strain R31, especially some peptidase, lipases, and glycoside hydrolases played a significant role in the predation process. The present study provides a new insight into the biocontrol of TBW and the recognition of myxobacteria predation against bacterial phytopathogen.

## Data Availability Statement

The original contributions presented in the study are included in the article/Supplementary Material, further inquiries can be directed to the corresponding author.

## Author Contributions

HD and AL designed the experiments. HD, XX, and RG performed the experiments. HD and YL analyzed the data. HD, QY, and HZ revised the manuscript. HD wrote the manuscript and responsible for the funding support. All authors read and approved the final manuscript.

## Conflict of Interest

The authors declare that the research was conducted in the absence of any commercial or financial relationships that could be construed as a potential conflict of interest.

## Publisher’s Note

All claims expressed in this article are solely those of the authors and do not necessarily represent those of their affiliated organizations, or those of the publisher, the editors and the reviewers. Any product that may be evaluated in this article, or claim that may be made by its manufacturer, is not guaranteed or endorsed by the publisher.

## Footnotes

1
https://www.ezbiocloud.net/

2
https://www.ncbi.nlm.nih.gov/nuccore/NZ_CP068048
