# Supplementary material for: Myxococcus xanthus R31 Suppresses Tomato Bacterial Wilt by Inhibiting the Pathogen Ralstonia solanacearum With Secreted Proteins
Source: Front Microbiol. 2022 Feb 7;12:801091. doi: 10.3389/fmicb.2021.801091 (PMC8859152; doi:10.3389/fmicb.2021.801091)
Supplement: Supplementary file 1 [file Data_Sheet_1.docx]

Supplementary Material

## Supplementary Figures


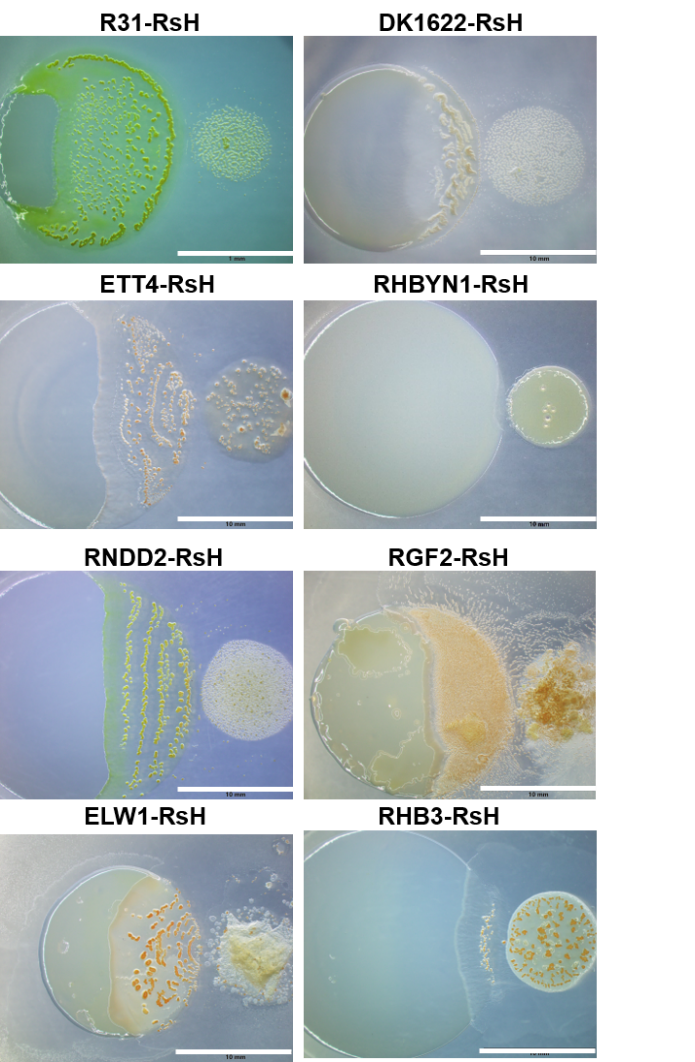


**Supplementary Figure 1.** Evaluated the predatory activity of the myxobacteria isolates against *R. solanacearum* RsH using plate experiment. The representative strains of seven species were selected for the experiment, and the model strain DK1622 was used as a control. 100 μL RsH cell suspension was pipetted onto the TPM plate and allowed to dry, and then 4 μL of myxibacteria suspension was spotted at 2 mm distance from the prey colony, and detailed experimental procedure was described in the Materials and Methods, scar bar = 5 mm; Triplicate experiments were performed.


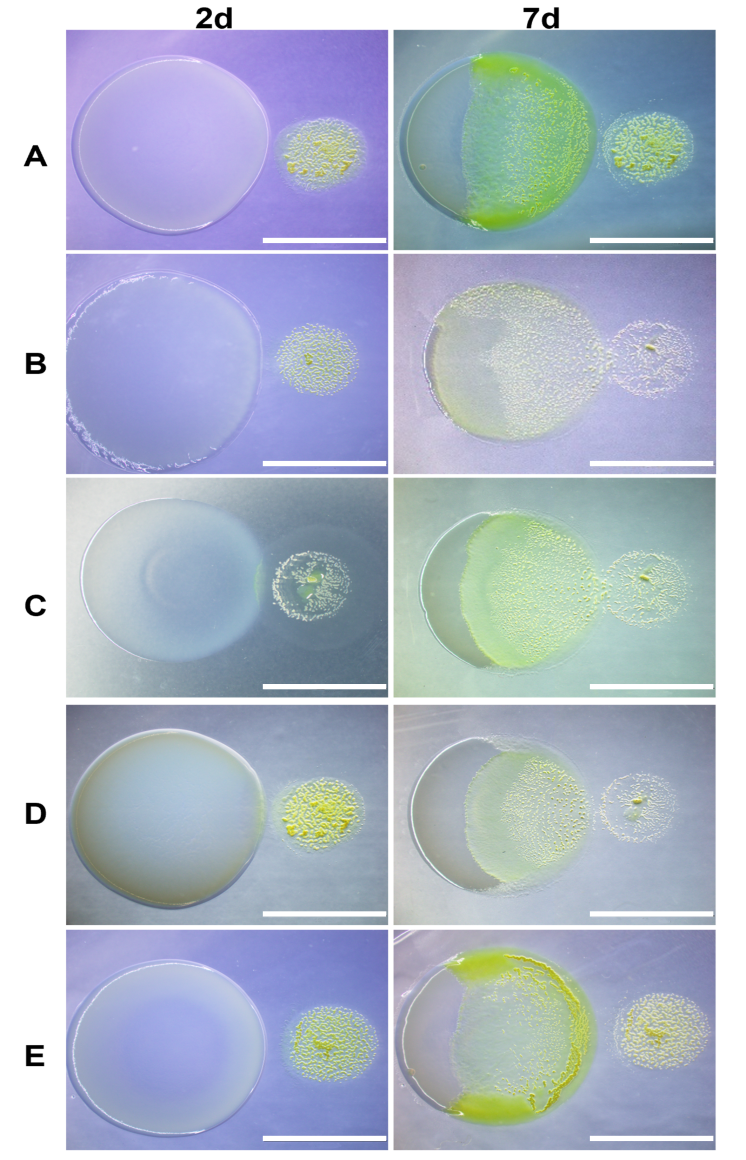


**Supplementary Figure 2.** Predation of strain R31 against *R. solanacearum* GIM 1.70 (**A**), *R. solanacearum* RS04 (**B**), *R. solanacearum* RsH (**C**), *R. solanacearum* GMI1000 (**D**) and *R. solanacearum* GIM1.335 (**E**). 100 μL *R. solanacearum* cell suspension was pipetted onto the TPM plate and allowed to dry, and then 4 μL of strain R31 suspension was spotted at 2 mm distance from the prey colony, and detailed experimental procedure was described in the Materials and Methods, scar bar = 5 mm; Triplicate experiments were performed.


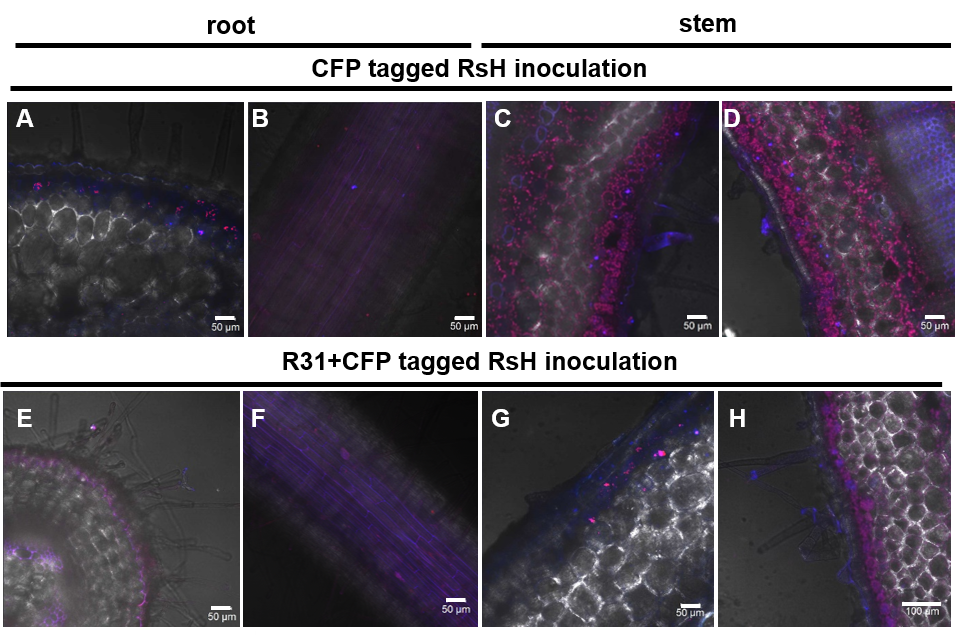


**Supplementary** **Figure 3.** Colonization of RsH on the root surface and stem of tomato seedlings as affected by inoculation of strain R31. Compared with ZN4 tomatoes only inoculated with RsH (**A-D**), simultaneous inoculated of stain R31 and RsH significantly decreased the abundance of RsH in tomatoes root (**E, F**) and stem (**G, H**) tissues. Scar bar = 50 μm for figure A- F, Scar bar = 100 μm for figure H. Triplicate experiments were performed.


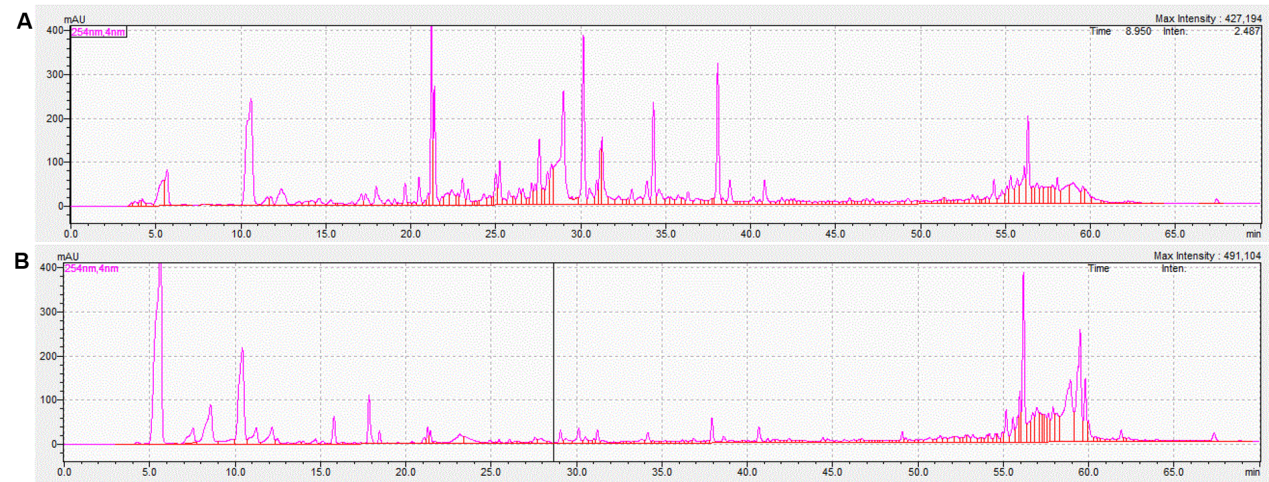


**Supplementary** **Figure 4.** High performance liquid chromatogram of strain R31 extraction product. (**A**) Fermentation broth ethyl acetate extract; (**B**) R31 strain ethyl acetate.


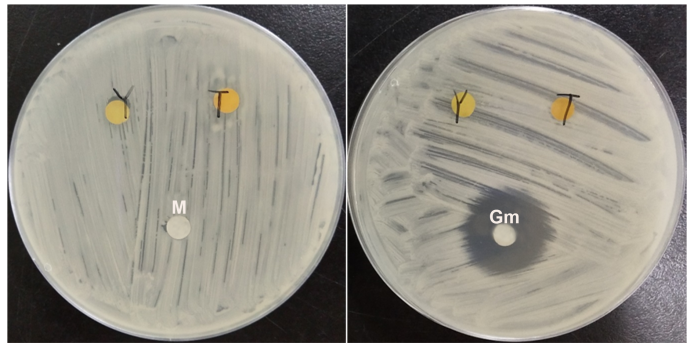


**Supplementary Figure 5.** The effects of strain R31 secondary metabolites for RsH. Y represents the extracellular secondary metabolite of strain R31, T represents the extracted intracellular secondary metabolite of strain R31, M represents methanol as a negative control, gentamycin (Gm) as a positive control.


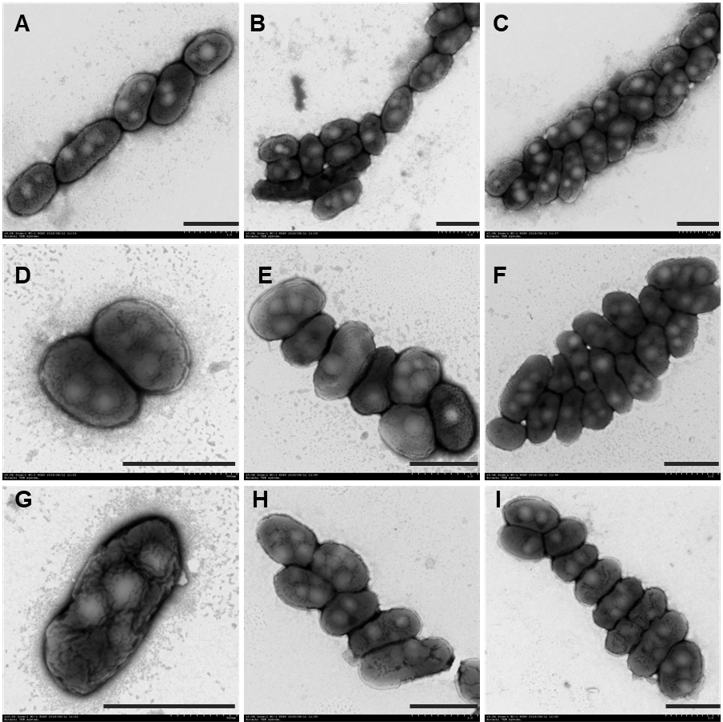


**Supplementary Figure 6.** Lysis of RsH cells by strain R31 extracellular proteins components. TEM micrograph of RsH cell after treated with 40%-60% saturation ammonium sulfate precipitated protein components (**A-C**), 60%-80% saturation ammonium sulfate precipitated protein components (**D-F**), and 80%-100% saturation ammonium sulfate precipitated protein components (**G-I**). Scar bar = 1 μm.
